# Trochanteric and subtrochanteric fractures irreducible by closed reduction: a retrospective study

**DOI:** 10.1186/s13018-023-03635-6

**Published:** 2023-02-26

**Authors:** Youliang Hao, Zhishan Zhang, Fang Zhou, Hongquan Ji, Yun Tian, Yan Guo, Yang Lv, Zhongwei Yang, Guojin Hou

**Affiliations:** 1 Department of Orthopaedics, Peking University Third Hospital, Beijing, 100191 China; 2 Engineering Research Center of Bone and Joint Precision Medicine, Beijing, 100191 China

**Keywords:** Trochanteric fractures, Irreducible, Preoperative radiographic features, Classification, Therapeutic strategies, Prognosis

## Abstract

**Aim:**

To explore the preoperative radiographic features and reduction methods of irreducible trochanteric and subtrochanteric fractures of the femur and to compare the perioperative characteristics and prognoses of irreducible and reducible fractures.

**Methods:**

The data of 1235 patients with femoral trochanteric fractures surgically treated in our hospital between January 2010 and January 2021 were retrospectively analyzed. According to the inclusion criteria and exclusion criteria, 1163 cases of femoral trochanteric and subtrochanteric fractures were included in this study. Fractures in which good or acceptable reduction could not be reached by closed manipulation were defined as irreducible fractures. The preoperative radiographic features, fracture displacement patterns after closed manipulation and intraoperative reduction methods used to treat irreducible fractures were analyzed, and the perioperative characteristics and prognoses of irreducible fractures and reducible fractures were compared.

**Results:**

There were 224 patients in the irreducible group and 939 patients in the reducible group. According to the radiographic features of fractures, irreducible fractures could be divided into four types: those with interlocking of the fracture, sagging of the femoral shaft, splitting of the lateral wall or medial wall, and comminution of the subtrochanteric area. Various kinds of reduction techniques were needed for different types.

**Conclusions:**

The incidence of irreducible trochanteric fractures was 15.4%, while the incidence of irreducible subtrochanteric fractures was 84.6%. According to the radiographic features of fractures, they can be divided into four types. It is important to identify irreducible fractures preoperatively and make comprehensive plans to the greatest extent possible to shorten the operation time, reduce intraoperative blood loss, and reduce the incidence of complications.

## Background

Trochanteric and subtrochanteric fractures account for approximately half of the fractures of the proximal femur [1]. These fractures are always treated surgically by closed reduction and internal fixation [2].

Great progress has been made in implant design for these fractures in recent decades [3–7]. Modern implants for intramedullary fixation allow immediate weight bearing in most cases. Despite these achievements in implant design, the complication rates are still very high [8–10]. Many factors are associated with complications, including the fracture pattern, bone quality, reduction quality, and implant placement [8]. However, reduction quality is the first and most essential factor that can be controlled by a surgeon. Longitudinal traction and internal rotation or external rotation of the fractured extremity will result in acceptable closed reduction of most fractures [11]. However, sometimes the fractures cannot be reduced by closed manipulation and require various kinds of open reduction techniques [12–16].

Although recent studies have reported some types of irreducible trochanteric fractures, they have only focused on irreducible pertrochanteric fractures, and the number of irreducible cases was relatively small [12–16]. The purpose of this study was to explore the preoperative radiographic features and reduction methods of irreducible trochanteric and subtrochanteric fractures.

## Methods

### Ethical approval

This study was conducted in accordance with the *Declaration of Helsinki* and was approved by the ethical committee of our university hospital. As the current study was retrospective in nature and data analysis was performed anonymously, this study was exempt from requiring informed consent from patients.

### Patient data

The medical records of 1235 patients who underwent surgery for trochanteric fractures at our institution between January 2010 and January 2021 were retrospectively reviewed. The inclusion criteria were as follows: (1) patients with a closed femoral trochanteric fracture who underwent surgery within 2 weeks after injury and (2) patients with complete clinical data, including radiographic examinations before and after surgery. The exclusion criteria were as follows: (1) patients with a pathologic fracture, delayed fracture, open fracture, or periprosthetic fracture, (2) patients with multiple fractures, (3) patients with developmental malformation of the femur, (4) patients with hip fusion caused by ankylosing spondylitis, and (5) patients with femoral head necrosis with obvious collapse. According to the inclusion criteria and exclusion criteria, 1163 cases of fractures were included in this study, with 1098 cases of trochanteric fractures and 65 cases of subtrochanteric fractures [17].

The quality of fracture reduction was described as *good*, *acceptable*, or *poor*, according to the modified criteria of Baumgaertner et al. [18] and Kim et al. [15] (Table 1). According to the reduction quality after closed manipulation, the patients were divided into reducible and irreducible groups. Those in whom *good* reduction or *acceptable* reduction was achieved were classified into the reducible group, while those in whom *poor* reduction was achieved were classified into the irreducible group.

**Table 1:** Quality of fracture reduction

1. Alignment [17]:
a. Anteroposterior view: normal or slightly valgus neck–shaft angle
b. Lateral view: less than 20° of angulation
2. Displacement of main fragments [14]:
a. Anteroposterior view: displacement less than the medial cortical thickness
b. Lateral view: displacement less than the anterior cortical thickness
Good, both criteria of alignment and both criteria of displacement
Acceptable, both criteria of alignment and only one criterion of displacement
Poor, only one or neither criterion of alignment or neither criterion of displacement

The preoperative radiographic features, fracture displacement patterns after closed manipulation, and intraoperative reduction methods of irreducible fractures were analyzed. The perioperative characteristics and prognoses of irreducible fractures and reducible fractures were compared. The perioperative characteristics included age, sex, length of hospital stay, time before the operation, operation time, and intraoperative blood loss.

### Operative protocol

Five experienced orthopedic surgeons performed all of the surgeries. Spinal anesthesia or general anesthesia was used. Reduction and internal fixation were performed with the patients in the supine position on a fracture table using an image intensifier. After closed manipulation, immediate intraoperative images were used to evaluate the reduction quality of the fracture. If the quality was good or acceptable, internal fixation was undertaken. If the fracture did not achieve good or acceptable reduction, limited open reduction techniques were performed; these included using a bone hook to pull the femoral shaft laterally, a clamp to reduce the fragments, a periosteum elevator to push the head–neck fragment and a Schanz screw as a joystick. Then, internal fixation was performed. The types of internal fixation used included an intramedullary fixation system (PFNA, PFNA-II, Gamma 3, Inter Tan) and an extramedullary fixation system (LISS, PFP, DHS).

### Follow-up method

Patients were asked to return to the hospital 1 month, 2 months, 3 months, 6 months, and 1 year after surgery. If the patients did not return on time, then a phone call or a video call was made to record the status of the patients. Patients who could not be reached after discharge were recorded as lost to follow-up. The Harris Hip Score (HHS) of the hip joint [19] was evaluated at every follow-up.

### Statistical analysis

The chi-squared test was used to analyze the distribution of categorical variables among groups for comparisons. For quantitative data, the one-sample Kolmogorov‒Smirnov test was used to test the normal distribution. Student’s t test or the Mann‒Whitney test was used to compare continuous variables as appropriate. All test results were considered significant when *P* < 0.05, and all statistical analyses were performed using SPSS 22.0 (SPSS, Chicago, IL, USA).

## Results

There were 939 patients in the reducible group and 224 patients in the irreducible group. In the irreducible group, there were 169 cases of trochanteric fractures and 55 cases of subtrochanteric fractures. The incidence of irreducible trochanteric fractures was 15.4%, while the incidence of irreducible subtrochanteric fractures was 84.6%. The differences between the two groups were statistically significant in terms of intraoperative blood loss and operation time (*P* < 0.001, Table 2).

**Table 2:** Preoperative and intraoperative data between the reducible and irreducible groups

	Reducible group *N* = 939	Irreducible group *N* = 224	*P* value
Age	78 (60–83)	77 (20–97)	0.079
Sex			0.052
Male	349 (37.2%)	99 (44.2%)	
Female	590 (62.8%)	125 (55.8%)	
Hospital stay (days)	6 (3–9)	7 (5–10)	0.068
Time before the operation (days)	4 (2–6)	4 (2–6)	0.599
Operation time (min)	64 (51–85)	126 (90–165)	< 0.001
Intraoperative blood loss (ml)	50 (50–100)	200 (100–400)	< 0.001

In total, 944 patients were followed up, while 219 were lost to follow-up. Moreover, 847 patients were followed up for more than 1 year.

The nosocomial mortality of the reducible group was higher than that of the irreducible group; however, the difference was not statistically significant (*P* = 0.849). The mortality rate within 1 year after surgery in the reducible group was higher than that in the irreducible group, but the difference was not statistically significant (*P* = 0.157). The malunion rate in the irreducible group was higher than that in the reducible group (*P* = 0.007). The rates of fracture nonunion and implant failure in the irreducible group were higher than those in the reducible group (*P* < 0.001, Table 3).

**Table 3:** Comparison of the prognoses of 944 patients who were followed up

Prognosis	Reducible group (*n* = 741) *N* (%)	Irreducible group (*n* = 203) *N* (%)	*P* value
Hospital death	7 (0.9%)	1 (0.5%)	0.849
Death within 1 year of surgery	58 (7.8%)	10 (4.9%)	0.157
Malunion	5 (0.7%)	7 (3.4%)	0.007
Nonunion and implant failure	11 (1.5%)	20 (9.9%)	< 0.001

The difference of median length of follow-up between the two groups was not statistically significant (*P* = 0.060). At the last follow-up, the difference of the HHS [19] between the two groups was not statistically significant (*P* = 0.516, Table 4).

**Table 4:** The HHS of patients followed up more than 1 year between the reducible group and irreducible group

	Reducible group (*n* = 672)	Irreducible group (*n* = 175)	*P* value
Follow-up time (months)	24 (12–40)	18 (12–36)	0.060
HHS at the last follow-up	80.00 (71.00–87.00)	82.00 (67.00–86.00)	0.516

### Types of irreducible fractures

**Fig. 1:**
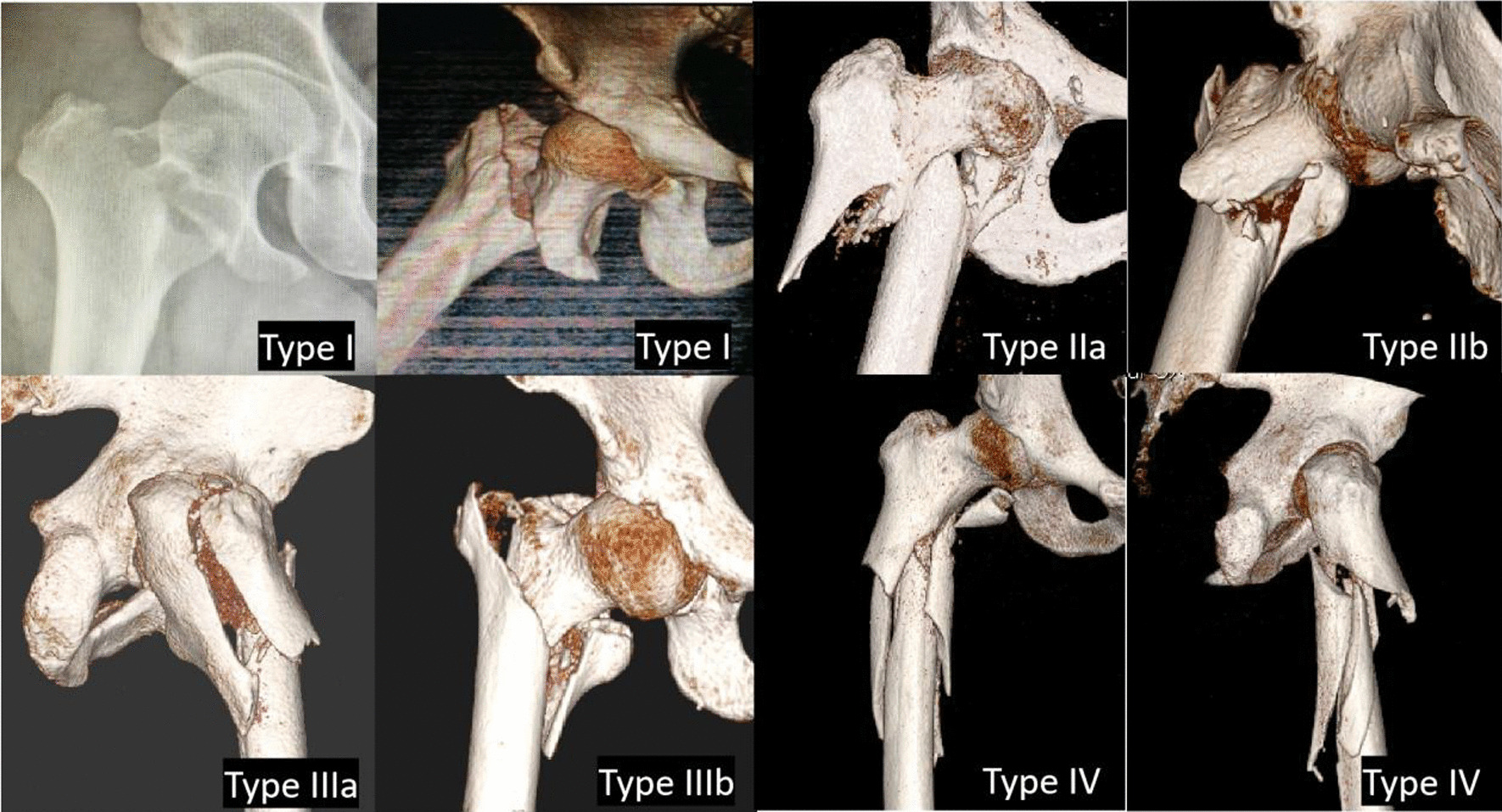
Type I: Interlocking of the fracture with the lesser trochanter bisected. Type IIa: Sagging of the femoral shaft without flection of the head–neck fragment. Type IIb: Sagging of the femoral shaft with flexion and anterior displacement the head–neck fragment. Type IIIa: Splitting of the lateral wall. Type IIIb: Splitting of the medial wall. Type IV: Comminution of the subtrochanteric area

According to the preoperative and intraoperative radiographic features, mechanism, and reduction methods used to treat fractures, irreducible fractures can be divided into four types (Fig. 1, Table 5).

**Table 5:** Median age and sex distribution between different types of irreducible fractures

	Type I *N* = 19	Type IIa *N* = 93	Type IIb *N* = 77	Type IIIa *N* = 12	Type IIIb *N* = 10	Type IV *N* = 13
Age	62 (20–92)	68 (30–97)	80 (61–97)	69 (45–90)	74 (44–87)	50 (23–83)
Sex						
Male	13 (68.4%)	44 (47.3%)	16 (20.8%)	7 (58.3%)	6 (60.0%)	13 (100%)
Female	6 (31.6%)	49 (52.7%)	61 (79.2%)	5 (41.7%)	4 (40.0%)	0

#### Type I

**Fig. 2:**
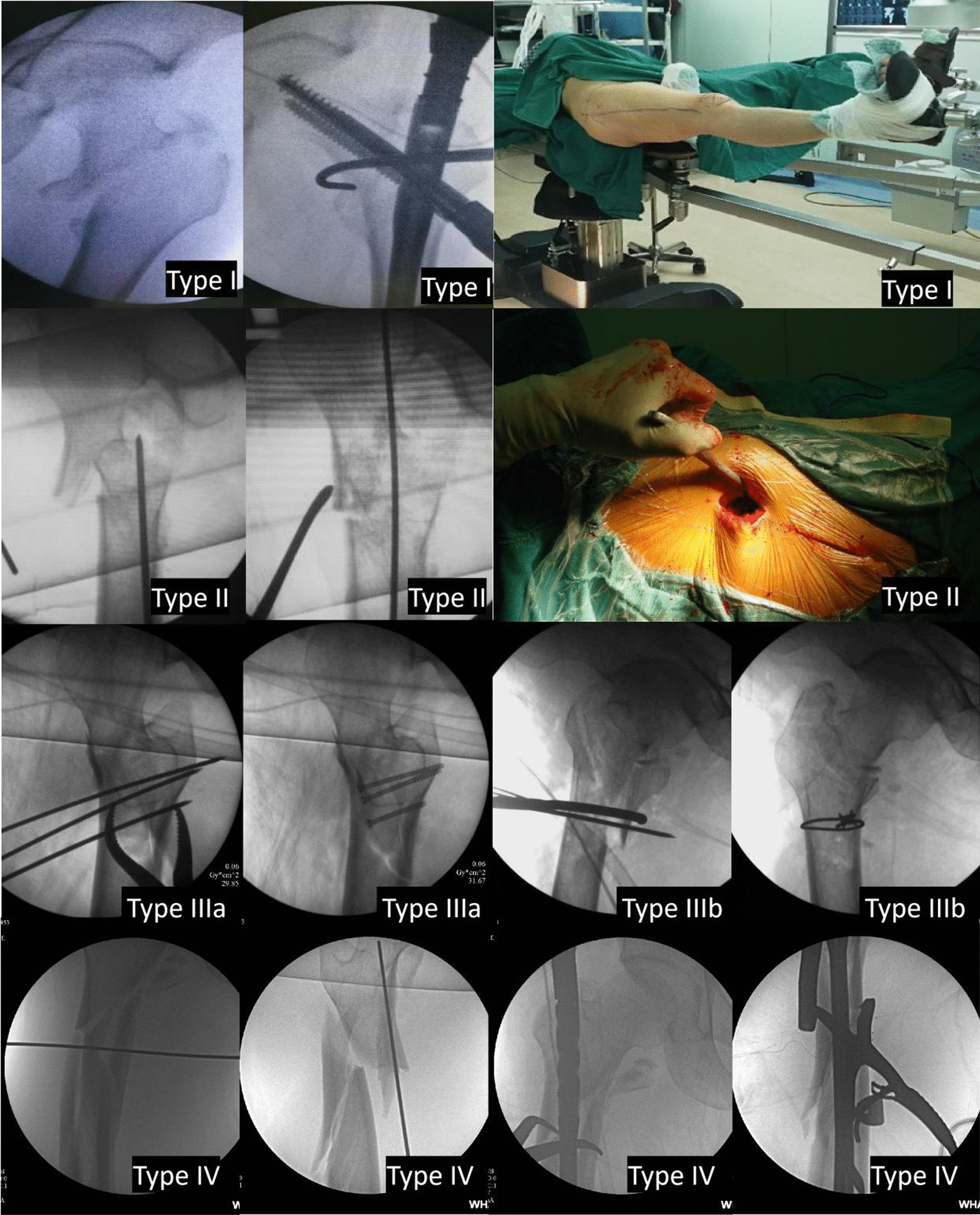
Reduction methods of irreducible trochanteric and subtrochanteric fractures

A similar radiographic feature, which involved a simple fracture line that ran from the proximal greater trochanter to the distal lesser trochanter, was seen in these patients. However, the lesser trochanter was bisected. Most of the greater trochanter was attached to the proximal head–neck fragment. The proximal fragment and distal fragment showed interlocking of each other and could not be unlocked by closed manipulation. The irreducibility of the fracture was due to interlocking by the iliopsoas muscles. First, to relax the iliopsoas muscles, we needed to reduce the traction force and then adduct and externally rotate the limb. Second, we used a bone hook to pull the head–neck fragment laterally and anteriorly. Third, we used Kirschner wires to provisionally fix the fracture. Finally, the fracture was fixed using an intramedullary fixation device (Fig. 2. Type I).

#### Type II

The preoperative radiographic feature of this type, which included 170 cases, was sagging of the femoral shaft. These fractures were divided into the following two subtypes according to the preoperative radiographic features.

#### Type IIa

A similar radiographic feature, which showed a reverse oblique or a transverse fracture line with posterior sagging of the femoral shaft, was seen in these patients. For this pattern, we tried to elevate the thigh with a mallet, which was successful in most of the cases. Occasionally, it was difficult to achieve good reduction with the mallet because we could not control the femoral shaft precisely. Then, we used a Schanz screw as a joystick to lift the femoral shaft.

#### Type IIb

A similar radiographic feature, which showed a fractured and medially displaced lesser trochanter and an evident flexion of the proximal fragment with an underlying lesser trochanter, was seen in these patients. The proximal fragment showed flexion and anterior displacement, while the femoral shaft showed posterior sagging. Longitudinal traction and rotation of the limb could not achieve acceptable reduction, so limited open reduction was then considered. A periosteum elevator was used to push the head–neck fragment posteriorly. A mallet was used to lift the femoral shaft. Keeping the periosteum elevator and mallet in situ, the fracture was fixed using an intramedullary fixation device (Fig. 2. Type II).

#### Type III

The preoperative radiographic feature of this type was splitting of the lateral wall or medial wall. These fractures were divided into the following two subtypes according to the preoperative radiographic features.

#### Type IIIa

A similar radiographic feature, which involved a coronal fracture line of the lateral femoral wall on the sagittal plane, was seen in these patients. These fractures showed good contact of the medial cortices and anterior cortices. However, the lateral fluoroscopic image showed separation of the lateral femoral wall on the sagittal plane. For this type, if intramedullary fixation was used, the hip screw would pass through the coronal fracture line of the lateral wall, which might aggravate the displacement of the lateral wall. Sometimes, extramedullary fixation could be an alternative method. For fractures with evident displacement of the lateral femoral wall, we tried to use a clamp to reduce the fragments and cannulated screws to fix them (Fig. 2. Type IIIa).

#### Type IIIb

A similar radiographic feature, which involved a coronal fracture line of the medial wall on the sagittal plane with an evidently displaced posteromedial fragment, was seen in these patients. A limited open reduction was considered, where a forceps was used to reduce the posteromedial fragment and titanium cables were used to fix the fragment. Then, the head–neck fragment and the femoral shaft were reduced and fixed following a general procedure (Fig. 2. Type IIIb).

#### Type IV

The preoperative radiographic feature of this type was comminution of the subtrochanteric area. A similar radiographic feature, which showed comminution of the subtrochanteric area with a sagging femoral shaft, was seen in these patients. For this type, a comprehensive reduction method, which included the use of bone hooks, reduction forceps, titanium cables, mallets to lift the femoral shaft, and other reduction techniques, might be used (Fig. 2. Type IV).

## Discussion

In the past 2 decades, several studies have been conducted on irreducible trochanteric fractures, with the incidence varying from 3.6 to 22.9% [12, 14, 16, 20, 21]. Moehring et al. [12] first described four patients with intertrochanteric fractures that were irreducible by usual closed manipulation and traction techniques. In our retrospective case series, 19.3% of cases were identified as irreducible by usual closed manipulation. Based on the preoperative and intraoperative radiographic features, mechanism, and reduction methods used to treat fractures, irreducible fractures could be divided into four types.

Type I fractures involving a simple interlocking fracture between the trochanters have been reported in some studies [12–14]. The irreducible mechanism of this type was that part of the iliopsoas tendon was attached to the proximal fragment and caused it to flex, while part of the iliopsoas was attached to the distal portion of the lesser trochanter, thus locking the proximal fragment in between. Sectioning of the iliopsoas tendon and extrication of interposed soft tissue are necessary to achieve a satisfactory reduction [12–14]. In this study, the reduction technique used was to reduce the traction, externally rotate the limb, and release the remaining attachment of the iliopsoas from the distal fragment. Then, the flexed proximal fragment could be reduced using a bone lever.

The preoperative radiographic feature of the second type was sagging of the femoral shaft. Sharma et al. [14] reported 12 cases of irreducible fracture similar to type IIb fractures in this study. The imaging of those fractures showed anterior flexion of the head–neck fragment, with an underlying separate lesser trochanter. Even after applying traction, the anterior flexion of the proximal fragment persisted. An underlying lesser trochanter fragment (usually large) seen in these fractures probably affects an anterior displacement of the proximal fragment, the lesser trochanter fragment itself being flexed by the pull of the iliopsoas. A lateral incision was suggested, and the fracture was reduced using a bone clamp or a bone lever. Similar cases were reported by some studies, and the reduction methods used were also similar [16, 20, 22]. A Steinmann pin or periosteum elevator was used to push the anterior cortex downward, while a hammer was used to lift the thigh. 

The preoperative radiographic feature of the third type was splitting of the lateral wall or medial wall. To the authors’ knowledge, few studies have been reported on this type of irreducible fracture. Type IIIa involved a coronal fracture line of the lateral femoral wall on the sagittal plane. These fractures showed good contact of the medial cortices and anterior cortices. However, the lateral fluoroscopic image showed separation of the lateral femoral wall on the sagittal plane. Gotfried [23] defined the lateral femoral wall first and reported that proximal femoral fractures with a fracture of the lateral femoral wall have high rates of implant failure. Palm et al. [24] reported that patients with a fracture of the lateral femoral wall had a sevenfold greater risk of reoperation following dynamic hip screw fixation than patients with an intact lateral femoral wall. Based on the authors’ experience, if intramedullary fixation was used, the hip screw would pass through the coronal fracture line of the lateral wall, which might aggravate the displacement of the lateral wall. Sometimes, extramedullary fixation could be an alternative method. Type IIIb involved a coronal fracture line of the medial wall on the sagittal plane with an evidently displaced posteromedial fragment. Both Evans classification [25] and AO/OTA classification [17] classify fractures without medial support as unstable. Many studies have shown that the continuity of the posteromedial cortex determines fracture stability, and reconstruction of medial support is the key to the treatment of unstable femoral trochanteric fractures [26–30]. In this study, a limited open reduction method was considered, where a forceps was used to reduce the posteromedial fragment and titanium cables were used to fix the fragment. Then, the head–neck fragment and the femoral shaft were reduced and fixed following a general procedure.

The preoperative radiographic feature of the fourth type was comminution of the subtrochanteric area. Biber et al. [11] reported that the displacement of subtrochanteric fractures varied, and reduction maneuvers depended on the patterns of displacement. However, less invasive methods are advisable to minimize soft tissue devascularization. According to this study, a comprehensive reduction method, which includes the use of bone hooks, reduction forceps, titanium cables, mallets to lift the femoral shaft, and other reduction techniques, might be used.

This categorization of four types may help surgeons to recognize irreducible fractures, make surgical plan, prepare reduction instruments, and shorten the operation time. This study had some limitations. First, this was a retrospective study, which makes it inherently more susceptible to missing data, bias, and confounding factors than a prospective study. Second, limited by the sample size, this study may not include all types of irreducible trochanteric fractures. We believe that this research must be continued by collecting a prospective cohort of patients to examine different treatment options and to study the adverse event incidences between the different types.

## Conclusions

The incidence of irreducible trochanteric fractures was 15.4%, while the incidence of irreducible subtrochanteric fractures was 84.6%. According to their radiographic features, irreducible fractures can be divided into four types: interlocking of the fracture, sagging of the femoral shaft, splitting of the lateral wall or medial wall, and comminution of the subtrochanteric area. It is important to identify irreducible fractures preoperatively, evaluate the perioperative risk, cross-match preoperatively, and prepare special reduction instruments to the greatest extent possible to shorten the operation time, reduce intraoperative blood loss, and reduce the incidence of complications.
